# The Cantor Lectures on Alcohol

**Published:** 1875-05-01

**Authors:** 


### THE CANTOR LECTURES ON ALCOHOL.

*From the London Lancet, April,* 1875.

THE last of six Cantor Lectures on the subject of Alcohol, its action, and its uses, was delivered by Dr. Richardson before the Society of Arts, on the 1st of February. Cantor, after whom the lectureship is named, was a member of the profession of physic, and left what may be called an Endowment to the Society of Arts, to be expended on some one or other of the useful purposes of that active and eminently serviceable public body. This session it was determined by the Council of the Society to invite public attention to the subject of alcohol and its influence on the health of the community. To be informed what effects alcohol produces on animal life was the object had in view; and, to carry this out, the council sought naturally for their lecturer a member of our body who, belonging to no particular community of reformers, and pledged to the support of no sectarian advocacy, should be able to speak with authority.

That the Society has in this measure taken a correct view is proved by the great interest that has been excited by the course of lectures now concluded. The subjects discussed have little direct reference to politics. Except in the opening sentences of the first lecture, when the lecturer touched on the one hundred and seventeen millions of money invested in the trade of alcohol in the United Kingdom, all political allusion was studiously avoided. The physician, he said, equally with the prelate and legislator, has a claim to be heard on a matter which essentially concerns the vital interests of the people; but, as the physician must restrict himself to the physiological side of the question, he cannot expect to receive the same degree of general attention as falls to the lot of more popular and favored speakers. The result has possibly for once corrected this expectation.

The subjects that have been prominently brought forward in the lectures include many in which the profession as well as the public feel an interest. The first lecture was an historical picture of the history of wine and spirit, of the origin of the processes of distillation in the laboratories of the experts of the Middle Ages, and of some additional notes on the general uses to which wine and its spirit have been applied in the service of man. The second lecture treated on the position of common or ethylic alcohol in the series of alcohols, and on the physiological action of the heavier alcohols, butylic and amylic. The third lecture described the primary physiological action of ethylic alcohol. The fourth was devoted to the question, Is alcohol food? The fifth and sixth directed attention to the physical deteriorations and mental alienations which follow the systematic and excessive use of alcoholic spirit.

The argument of the lecturer on the question advanced in the fourth lecture—Is alcohol food?—led to a negative answer, while at the same time it admitted the validity of the statement so powerfully enforced by the late Dr. Anstie, viz., that alcohol is disposed of in the living body by other processes than simple, direct elimination. This view, however, that alcohol is chemically changed within the organism, was not admitted as any proof in favor of the assumption that the substance becomes in any sense a food. The evidence was considered conclusive that alcohol is not a builder of any tissue whatever, probably not even of fatty tissue. It therefore either becomes a food as an agent which supplies force, or it is no food whatever. If, by oxidation, it be consumed like a heat-giving food, the evidence of the fact ought to appear in the experiment of its administration. Does the evidence appear? To the solution of this question Dr. Richardson brought to bear the many facts at his command. He re-affirmed the statement originally made by him, from experimental observation, in his report to the British Association at Birmingham in 1866, that the general effect of alcohol is to reduce the animal heat. In the first stage of alcoholic excitement the external temperature of the body is slightly raised, and in the confirmed inebriate this primary elevation of temperature may be distinctly marked. But the effect is held to be due, not to a greater combustion, but to a quicker loss of temperature. It is, in truth, a process of cooling; it is from the unfolding of the larger sheet of the warm blood in vessels partly paralyzed by the alcohol, and from the quicker radiation of heat from that larger surface. During this stage the internal temperature is declining, the expired air from the lungs is indicating the first period of reduction in the amount of carbonic acid, and the reddened surface of the body is so reduced in tone that cold applied to it increases the suffusion. It is this most deceptive stage that led the older observers into the error that alcohol warms the body. In the second and third stages the temperature declines, falling below the natural; and, as the fourth stage is approached, it reaches a decline that becomes actually dangerous. This reduction is marked in some degree in proportion to the depth of the coma that follows the intoxication. With the first conscious movements of recovery, there is a faint rise of temperature, but such is the depression that these very movements exhaust and lead to a further reduction. In extreme instances so long a period as three days is required to bring back a steady natural return of the animal warmth. Through every stage, therefore, except the first, the special action of alcohol is to reduce the animal temperature. In fact, cold and alcohol act in the same manner on the vital processes. Both paralyze the vessels of the minute circulation; both lead, therefore, at certain stages of their action, to suffusion of the surface of the body, and both reduce power. In proof of this, it was shown that if two warm-blooded animals are placed asleep in the same air, brought to a low degree of temperature, the one animal charged with alcohol, the other free of the agent, they are differently affected. They both sleep under these circumstances; but the alcoholic sleeps to die; the other simply sleeps more deeply than is natural, and lives so long as the store of food it is charged with continues to support life. Within this bond it awakes in a warmer air uninjured, though the degree of cold be carried even to the degree of freezing of the extreme parts. To this evidence is added the fact that the amount of carbonic acid excreted by the animal that is under alcohol is decreased.

From all these facts the inference is that the alcohol is not burned after the manner of a food which supports animal combustion, but that it is decomposed into secondary products, by oxidation, at the expense of the oxygen which ought to be applied to the natural heating of the body.

These conclusions respecting the action of alcohol on the animal temperature were backed up by another series of observations, in which the effect of this substance was traced on the muscular power. Here it was shown that just as the animal heat is reduced so is the excitability of muscle, until at last, as narcotism is developed by the spirit, the muscular power is brought down to the extreme of prostration.

The practical deductions from these observations accord with the modern experience that is now being gained under the new light by which this subject is surveyed. To the professional mind the facts have for some time past been gradually developing, and have afforded striking evidence of the value of properly conducted experimental research. To the public mind they are, however, new, and are so opposed to the preconceived ideas and the false experience of the past that they are startling in their novelty. So much the more important is it that the public should be correctly informed of the progress of medical science on matters which so greatly concern the general welfare. Legitimate medicine never flourishes so healthily as when an intelligent public appreciation of its labors and its knowledge is manifested and encouraged. We have touched, on the present occasion, on one point only in these lessons communicated to the Society of Arts. We shall return to them in a future number.

